# Transhepatic Embolization of Congenital Intrahepatic Portosystemic Venous Shunts with Associated Aneurysms

**DOI:** 10.1155/2015/375086

**Published:** 2015-07-14

**Authors:** Kalyan Paudel, Eric K. Hoffer

**Affiliations:** Department of Vascular and Interventional Radiology, Dartmouth-Hitchcock Medical Center, Lebanon, NH 03766, USA

## Abstract

Intrahepatic shunts between the portal and systemic venous systems with associated aneurysms are extremely rare. A middle aged woman presented with hepatic encephalopathy and was found to have two intrahepatic portosystemic venous shunts with associated aneurysms. Diagnosis was made by duplex ultrasound and was confirmed with contrast enhanced MRI. Treatment was performed percutaneously with an Amplatzer vascular plug.

## 1. Case Report

A 45-year-old woman presented with right upper quadrant abdominal pain, swelling, and symptoms consistent with encephalopathy (confusion, altered level of consciousness). Her RUQ pain had become more intense and continuous over the prior few months, prompting her to seek medical treatment. She had no history of fever, nausea, vomiting, or changes in bowel habits. No prior hepatic, renal, or cardiovascular diseases were reported. There was no history of abdominal surgery, trauma, liver biopsy, or alcohol abuse.

On physical examination, the patient was somnolent, but stable, with no signs of distress. There was slight swelling in the RUQ with mild tenderness. There was no hepatosplenomegaly, rebound tenderness, or rigidity. Bowel sounds were normal. Laboratory data including liver function and blood ammonia levels were within normal limits.

**Figure 1:**
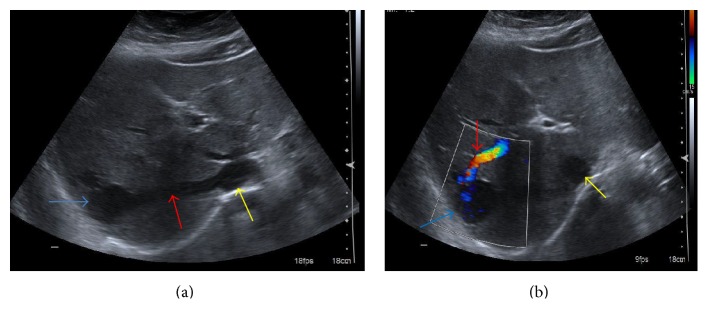
A 45-year-old female with congenital intrahepatic portosystemic venous shunts with associated aneurysms. (a) US image shows an abnormal communication between the right hepatic vein (red arrow) and right portal vein through an aneurysm (blue arrow). Inferior vena cava (yellow arrow) is also seen. (b) Color Doppler US image demonstrates the communication of the right portal vein (red arrow) to the right hepatic vein (not shown) through an aneurysm (blue arrow) within segment VII of the liver. Color Doppler reveals flow within the right portal vein (red arrow) and the aneurysm (blue arrow). Inferior vena cava (yellow arrow) is also seen. Technique: curvilinear 1–4.5 MHz transducer performed on a Siemens Acuson S3000 ultrasound machine.

*Imaging Workup*. Abdominal ultrasound (US) and color Doppler interrogation demonstrated communication between the right portal vein and the right hepatic vein through an aneurysm within segment VII of the liver (Figures 1(a) and 1(b)). The liver had a regular contour and homogeneous parenchyma. No ascites was noted.

**Figure 3:**
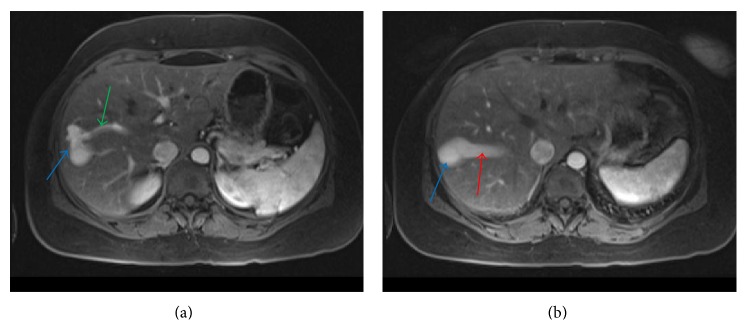
A 45-year-old female with intrahepatic portosystemic venous shunt with associated aneurysms. (a) Contrast enhanced axial VIBE MRI image showing communication between the right portal vein (green arrow) and right hepatic vein through an aneurysm (blue arrow). (b) Contrast enhanced axial VIBE MRI (a few sections cranial to Figure 2(a)) showing communication between the right portal vein and right hepatic vein (red arrow) through a second larger aneurysm (blue arrow). Protocol: Siemens, 1.5 Tesla Avanto MR Scanner, TR = 4.3, TE = 1.91, 3.5 mm slice thickness, Matrix = 192 × 256, with 15 mL Gadobenate dimeglumine (Multihance, Bracco Diagnostics Inc.) injection.

**Figure 4:**
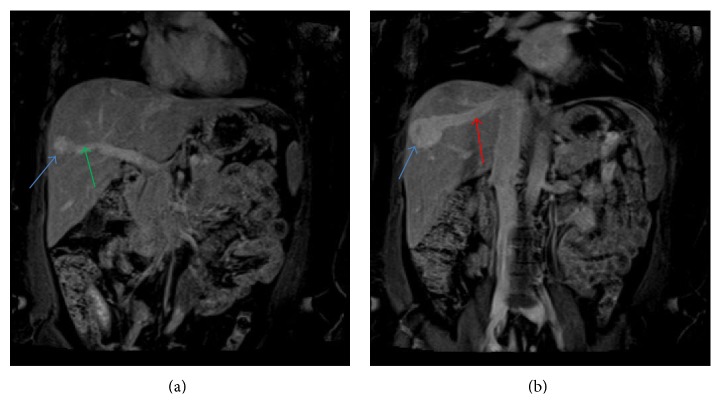
A 45-year-old female with intrahepatic portosystemic venous shunt with associated aneurysms. (a) Contrast enhanced coronal VIBE MRI showing communication between the right anterior portal vein branch (green arrow) and right hepatic vein through 28 mm aneurysm (blue arrow). (b) Coronal VIBE MRI (a few sections posterior to (a)) demonstrating communication between the right portal vein and right hepatic vein (red arrow) through a second, larger, 37 mm aneurysm (blue arrow). Protocol: Siemens, 1.5 Tesla Avanto MR Scanner, TR = 4.3, TE = 1.91, 3.5 mm slice thickness, Matrix = 256 × 256, with 15 mL Gadobenate dimeglumine (Multihance, Bracco Diagnostics Inc.) injection.

Multiplane, multisequence magnetic resonance imaging (MRI) of the abdomen with and without contrast revealed two intrahepatic portohepatic venous shunts, each associated with an aneurysm (Figures 3(a), 3(b), 4(a), and 4(b)). These were thought to be congenital in origin.

**Figure 5:**
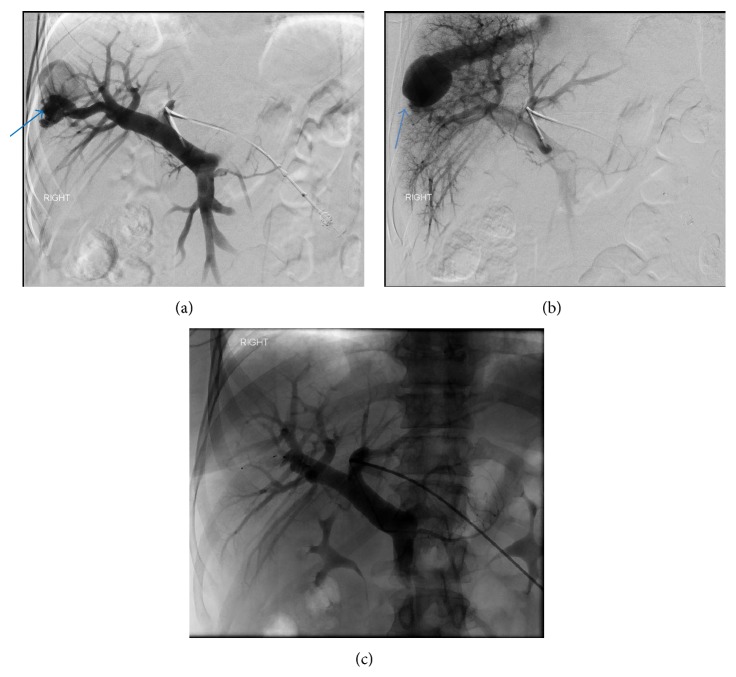
A 45-year-old female with intrahepatic portosystemic venous shunt with associated aneurysms. (a) Portal venography obtained via left portal vein with catheter tip in the main portal vein demonstrates a right anterior portal vein branch communicating with the right hepatic vein through a 28 mm aneurysm (arrow). (b) Portal venography obtained via left portal vein with catheter tip in the main portal vein shows a right posterior portal vein branch communicating with the right hepatic vein through a second, larger, 37 mm aneurysm (arrow). (c) Portal venography performed after embolization of both anterior and posterior right portal branches communicating with the right hepatic veins with Amplatzer vascular plugs demonstrates obliteration of intrahepatic portosystemic shunts. Protocol: Single Plane Artis Zee Siemens system, embolization with a 12 mm and 6 mm Amplatzer vascular plug (*St Jude Medical)*.

*Treatment*. Therapy was indicated due to persistent and progressive symptoms of encephalopathy. Although an open surgical approach was considered, the multidisciplinary liver tumor group elected an initial percutaneous interventional approach. The procedure was performed with intravenous moderate sedation and local anesthesia. The left branch of the portal vein was accessed with a 21-gauge needle from an anterior abdominal approach, under real time US guidance. After placement of a 10 cm 5 Fr vascular sheath, a 4 Fr Bernstein catheter (Cook, Inc., Bloomington, IL) was directed into the main portal vein over a 0.035-inch angled glide-wire (Terumo Medical Corp., Somerset, NJ). Portal venography demonstrated two large right portal anterior and posterior venous branches communicating with the right hepatic veins through aneurysmal connections (Figures 5(a) and 5(b)). There was a 28 mm aneurysm associated with the anterior portohepatic venous shunt (Figure 5(a)) and a 37 mm aneurysm associated with posterior portohepatic shunt (Figure 5(b)). Over a 0.035-inch Rosen wire, the 10 cm sheath was exchanged for a 25 cm 5 Fr sheath. The 4 Fr Bernstein catheter was advanced over the 0.035-inch angled glide-wire into the anterior right portal vein branch to its communication with the right hepatic vein. A 6 mm Amplatzer II vascular plug (St Jude Medical, St. Paul, MN) was used to occlude the right anterior portal vein proximal to the communication with the right hepatic vein. The 4 Fr Bernstein catheter was then directed to the posterior right portal vein branch that supplied the second shunt. A 12 mm Amplatzer vascular plug was placed to occlude the posterior right portal vein branch. After occlusion of anterior and posterior right portal vein branches, a portal venogram was obtained with catheter tip in the main portal vein, which confirmed an absence of flow in the occluded branches with no opacification of the aneurysms (Figure 5(c)). The hepatic parenchymal access tract was embolized with gelfoam pledgets.

The patient was admitted overnight for pain management and observation. Her moderate postprocedure RUQ pain was managed with intravenous hydromorphone. There was improvement of her RUQ pain and swelling at one-week postprocedure follow-up. US and Doppler assessment of the liver performed one month after embolization demonstrated thrombosis of the portohepatic communications and associated aneurysms. The patient had complete resolution of the presenting symptoms and was asymptomatic at 6-month follow-up.

## 2. Discussion

Stringer described four major different varieties of congenital portosystemic venous shunts in six children seen during a 10-year period, with reference to anatomy of the shunt which was determined by imaging studies and surgery [1]. He divided extrahepatic portocaval shunts into two types: first, end-to-side shunts, where the portal vein (PV) terminates in the inferior vena cava (IVC), and the second, side-to-side shunts in which there is venous communication between a patent PV and the IVC. The third and fourth types of intrahepatic portosystemic shunts were abnormal intrahepatic connections between branches of the PV and the hepatic veins or IVC and persistent patent ductus venosus.

An intrahepatic portosystemic venous shunt (IPSVS) is a communication between an intrahepatic portal vein and a systemic vein via an anomalous intrahepatic venous channel. The etiology of IPSVS may be congenital or acquired (secondary to cirrhosis, trauma, or biopsy procedure). The intrahepatic portal venous shunt in adults is most commonly due to portal venous communication to a systemic vein secondary to portal hypertension. While portosystemic shunts less than 2 mm in diameter are relatively common in patients with cirrhosis, the larger shunts are thought to be congenital [2]. Popper et al. [3] described many minute communications between the portal and hepatic veins in cirrhotic livers. These anastomoses were considered to be remnants of previous sinusoids that had dilated and whose walls had become thickened in areas of liver cell loss [4]. Our case is likely to be congenital as these were relatively large communications and there was no associated evidence of chronic liver disease or prior history of trauma to the liver.

In the embryonic period, there are three pairs of veins that participate in the formation of venous structures within and around the liver: right and left vitelline veins, umbilical veins, and cardinal veins. The portal vein is formed from several segments of the vitelline veins while some segments of the right and left vitelline veins collapse and disappear during the process of development [5]. According to Raskin et al. [4] “a persistence of portions of the omphalomesenteric (vitelline) venous system from the second month of fetal life is the most likely explanation for the portohepatic venous malformation.”

Some small intrahepatic portosystemic shunts (Table 1) located between the portal branches and hepatic veins disappear spontaneously by age 1 to 2 years [6, 7]. If larger shunts or the ductus venosus persists, they pose a risk of late development of complications such as hepatic encephalopathy, pulmonary hypertension, or hyperammonemia [4, 6, 7]. The clinical significance of IPSVS depends upon the shunt ratio and on the patient's age. Decreasing tolerance of the brain to toxic metabolites with increasing age may explain the delayed clinical manifestations [4, 8].

Two shunts in one patient are also relatively rare. Remer et al. found three out of twenty-two (13.6%) had two intrahepatic portal venous shunts [9]. The incidence of portal vein aneurysm in association with portosystemic shunts is not uniform in the literature. Tanoue et al. found that portal vein aneurysms were reported in 5 (50%) of 10 cases [10].

Color Doppler ultrasonography (US) is the key imaging modality for the diagnosis of congenital portosystemic shunt [11]. Doppler US is also useful for follow-up after therapy. In addition to demonstrating flow signals between the involved vessels, and evaluating flow direction, it may also determine the shunt ratio by estimating flow volume [12, 13]. The shunt ratio is calculated by dividing the blood flow volume through the shunt by the total portal blood flow volume [6, 14]. When the shunt ratio increases, the amount of nitrogen-containing substances in the portal blood that bypass the hepatic metabolism rises in the systemic circulation and can lead to hepatic encephalopathy. When the shunt ratio exceeds 30%, hepatic encephalopathy may develop at any time [15]. When the shunt ratio exceeds 60%, the risk of hepatic encephalopathy is increased, such that, in noncirrhotic patients even without encephalopathy, therapeutic intervention is indicated [6, 14].

Multidetector computed tomography (CT) with contrast is useful for pretreatment planning, to document the anatomy and location of the shunt. With use of maximum intensity projection and multiplanar reconstruction, it provides all the necessary information about the course of the shunt, its size, and orientation; it helps to define the best access route for intervention [16]. MRI with contrast of the abdomen can also well visualize the shunt anatomy without radiation risk. Confirmation with CT or MRI is valuable when there is suboptimal sonographic visualization of the liver and can exclude associated abnormalities such as liver tumors.

Recently, Lautz et al. classified shunts based on portal anatomy and expected physiologic consequences of the shunt (Table 2), regardless of whether they were intrahepatic or extrahepatic; type I shunts were those with no intrahepatic portal flow and type II shunts were partial shunts with some preserved intrahepatic flow [18]. Type II shunt was further classified based on portal anatomy; type IIa arises from a portal branch, type IIb is from the main portal vein, its bifurcation, or the splenomesenteric confluence, and type IIc includes shunts from mesenteric veins [18].

Other vascular abnormalities of the liver such as cavernous transformation of the portal vein, arterioportal shunts, and aneurysm of the portal vein are in the differential diagnosis. These can be differentiated from IPSVS by imaging findings. Cavernous transformation of the portal vein is due to the formation of venous channels within and around a previously stenosed or occluded portal vein that act as portoportal collateral channels [17]. A characteristic beaded appearance (mass of veins) at the porta hepatis is usually seen on contrast enhanced CT scans due to dilated biliary and gastric veins [18–20]. Arterioportal shunts are due to communication between the hepatic artery and the portal venous system. This condition is diagnosed in helical CT performed during the hepatic arterial phase showing early and marked enhancement of the main portal vein or major tributaries with attenuation similar to that of the aorta [17]. Aneurysms of the portal venous system may be present in patients with liver disease [17]. On contrast enhanced CT or MRI, an aneurysm appears as an enhancing cystic mass that arises from the portal venous system and demonstrates simultaneous enhancement with portal vein.

Invasive studies such as arterial portography or direct percutaneous transhepatic portography can confirm the presence of portosystemic shunts [17]. Symptomatic portosystemic shunts warrant therapeutic intervention. Treatment options include percutaneous embolization or surgical shunt ligation and/or hepatic resection. Liver transplantation is the only curative treatment of congenital absence of the portal vein (type I Abernethy malformation) and is reserved for patients with refractory symptoms despite medical management [7, 18]. Type II PSVS has variable anatomy, clinical features, and treatment options [18]. Lautz et al. reported 10 symptomatic children with type II PSVS who were successfully managed with operative ligation (*n* = 6), endovascular occlusion (*n* = 3), and a combined approach (*n* = 1). Endovascular occlusion (using coils, plugs, and/or stents) has proven successful for patent ductus venosus (PDV) and other isolated type IIa shunts whereas type IIb shunt usually requires staged operative closure because of short and wide diameter of the shunt [18]. Type IIc can be managed either with surgical ligation or endovascular occlusion [18]. Percutaneous closure of the shunts can be performed when an occlusive device can be fixed in position in the shunt, without compromising the flow in the inferior vena cava and the normal hepatic veins [11]. This endovascular therapeutic option applies to shunts between portal branches and hepatic veins that can be closed by means of Amplatzer occlusive devices or embolic metallic coils, which depends on the size, position, and number of communications [11]. Advantages of percutaneous embolization are the less invasive procedure and preservation of hepatic parenchyma. This is particularly important when faced with a lesion in the setting of cirrhosis and portal hypertension where preservation of hepatic parenchyma is desired [21].

After closure of the shunt, liver tests and coagulation studies should return to normal. Hyperammonemia usually normalizes within a day and serum bile acids should be normal after a few days [10, 22]. Sustained clinical follow-up is necessary to exclude return of symptoms that are secondary to recanalization of an occluded shunt or enlargement of other anomalous portosystemic communications.

## 3. Conclusion

Congenital intrahepatic shunts between the portal and hepatic veins are rare vascular abnormalities that may cause hepatic encephalopathy. Abdominal sonography with color Doppler is an excellent method to identify these lesions. Confirmation with CT or MRI is valuable when there is suboptimal sonographic visualization of the liver, to identify associated abnormalities, and for treatment planning. This pathologic condition can be successfully treated with percutaneous interventional occlusive techniques or surgical closure.

## Figures and Tables

**Figure 2:**
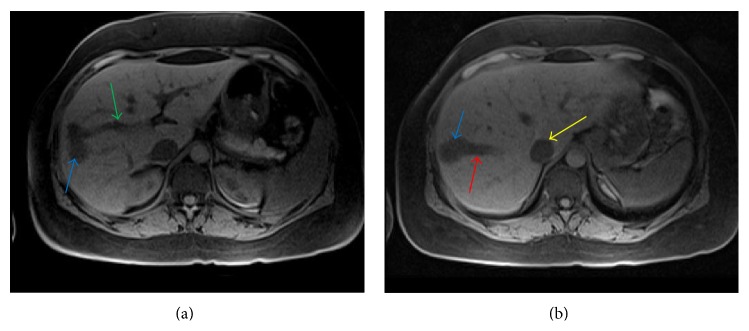
A 45-year-old female with intrahepatic portosystemic venous shunt with associated aneurysms. (a) Noncontrast axial VIBE MRI image showing communication between the right portal vein (green arrow) and right hepatic vein through an aneurysm (blue arrow). (b) Noncontrast axial VIBE MRI image (a few sections cranial to (a)) demonstrating communication between the right portal vein and right hepatic vein (red arrow) through a second larger aneurysm (blue arrow). Protocol: Siemens, 1.5 Tesla Avanto MR Scanner, TR = 4.3, TE = 1.91, 3.5 mm slice thickness, Matrix = 192 × 256, no contrast.

**Table 1:** Summary of intrahepatic portosystemic shunts.

Etiology	Intrahepatic portosystemic shunts can be acquired (secondary to cirrhosis, trauma, or biopsy procedure) or congenital.

Incidence	Age of presentation young to middle age.

Gender	Both males and females are affected.

Treatment	Endovascular treatment (occlusion with coil or Amplatzer vascular plug), surgical ligation, or resection.

Prognosis	May be asymptomatic. Can resolve spontaneously. If symptomatic, there is good prognosis with treatment.

Findings on imaging	Doppler US: presence of vascular structures connecting a portal branch to a hepatic vein with or without aneurysm formation. Undulating triphasic waveform pattern in the portal vein similar to hepatic waveform.
	CT/MRI with contrast: presence of vascular structures connecting a portal branch to a hepatic vein with or without aneurysm formation.

**Table 2:** Classification of congenital PSVS [17]. CAPV indicates congenital absence of the portal vein.

Type	Description
I	No intrahepatic portal flow (CAPV or type I Abernethy malformation)
II	Partial shunt with preserved hepatic portal flow (type II Abernethy malformation)
IIa	Arising from left or right portal vein (including PDV)
IIb	Arising from main portal vein (including its bifurcation or splenomesenteric confluence)
IIc	Arising from the mesenteric, gastric, or splenic veins

## Abbreviations

CT: Computed tomography
CAPV: Congenital absence of the portal vein
Fr: French scale commonly used to measure the size of a catheter
IPSVS: Intrahepatic portosystemic venous shunt
IVC: Inferior vena cava
PDV: Patent ductus venosus
PSVS: Portosystemic venous shunt
MRI: Magnetic resonance imaging
RUQ: Right upper quadrant.
